# The Microbiota Dynamics of Alfalfa Silage During Ensiling and After Air Exposure, and the Metabolomics After Air Exposure Are Affected by *Lactobacillus casei* and Cellulase Addition

**DOI:** 10.3389/fmicb.2020.519121

**Published:** 2020-11-26

**Authors:** Zongfu Hu, Huaxin Niu, Qing Tong, Jie Chang, Jianhua Yu, Shuguo Li, Shi Zhang, Deying Ma

**Affiliations:** ^1^College of Animal Science and Technology, Northeast Agricultural University, Harbin, China; ^2^College of Animal Science and Technology, Inner Mongolia University for Nationalities, Tongliao, China

**Keywords:** alfalfa silage, epicuticular wax, microbiota dynamics, lactic acid bacteria, metabolomics, cellulase, *Lactobacillus casei*

## Abstract

Both inoculants treatment and enzyme treatment promote the reproduction of lactic acid bacteria (LAB) to produce enough lactic acid to lower pH in silage. The present study investigated the microbial community and metabolome in cellulase, *Lactobacillus casei*, and air treated alfalfa silage. Chopped and wilted alfalfa (first cutting, 29% dry matter) was ensiled without (CON) or with *L. casei* (1 × 10^6^ cfu g^–1^ fresh matter) (LC) or cellulase (20,000IU, 0.5% of fresh matter) (CE) for 56 days, then exposed to air for 3 days (PO). Greater ensiling quality was observed in LC and CE, which had lower pH and higher lactic acid content than CON at 56 days of ensiling and 3 days post-oxygen exposure. Air exposure was associated with decreased lactic acid concentrations and increased yeast and mold counts in all silages. SEM showed that the structure of leaf epicuticular wax crystals were intact in fresh alfalfa, totally decomposed in CON silage, and partly preserved in CE and LC silage. Gas chromatography mass spectrometry revealed that 196 metabolites and 95 differential concentration were present in the 3 days air exposure samples. Most of these metabolites, mainly organic acids, polyols, ketones, aldehydes, are capable of antimicrobial activity. The bacterial communities were obviously different among groups and *Lactobacillus* developed to a dominant status in all silages. *Lactobacillus* became dominant in bacterial communities of LC and CE silages from days 7 to 56, and their relative abundances reached 94.17–83.93% at day 56, respectively. For CON silage, until day 56, *Lactobacillus* dominated the bacterial community with abundance of 75.10%. After 3 days of oxygen exposure, *Lactobacillus* and *Enterococcus* were predominant in CON, and *Lactobacillus* remained dominant in LC and CE silages. The results indicated that, compared to untreated silages, *L. casei* could be a priority inoculant for alfalfa silage to boost *Lactobacillus* abundance and improve fermentation quality. Our high-throughput sequencing and gas chromatography mass spectrometry results provide a deep insight into the bacterial community and metabolites in alfalfa silage.

## Introduction

Alfalfa is an important legume used as forage in animal husbandry, but is more difficult to ensile than other forages due to its high buffering capacity, low water-soluble carbohydrate (WSC) content and a tubular hollow stem that inhibits the complete removal of air during ensiling (McAllister et al., 1998; Nkosi et al., 2016). Ensiling is an effective method for maximizing the preserved nutritive value of forage crops or grasses (Pahlow et al., 2003). The improvement of fermentation quality of silages, evaluated by low pH, high lactic acid content, and low level of ammonia-N, can be achieved by treatment of silage with different additives, including inoculants, chemical matter and enzymes (Dunière et al., 2013; Ni et al., 2017; Muck et al., 2018).

Lactic acid bacteria (LAB) are extensively used in silage for their ability to accelerate the accumulation of lactic acids, lower pH, prevent protein degradation, reduce dry matter (DM) loss, and enhance animal performance (Weinberg and Muck, 1996; Filya et al., 2006; Oliveira et al., 2017). Unlike *L. plantarum* and *L. buchneri*, which conserve forage (Arriola et al., 2011; Contreras-Govea et al., 2011), *L. casei* (*L. paracasei*) has not frequently been used in silage, as its effects are uncertain. Some studies reported a positive effect of *L. casei* on silage, such as increased lactic acid, and decreased acetic acid (Nishino et al., 2007; Nishino and Touno, 2010). However, other studies have revealed possible negative effects of *L. casei* on silage, which found that *L. casei* was present in poor quality alfalfa silages with low acid content, which are characteristics of low quality silage (Langston and Bouma, 1960).

Enzymes can increase the rate of plant cell wall degradation by hydrolyzing cellulose fibers to soluble carbohydrates (Muck et al., 2018). The release of soluble carbohydrates promotes the growth of LAB, which produce lactic acid and decrease the pH of silage (Kung et al., 1991; Nadeau et al., 1996; Su et al., 2019). Conversely, Lynch et al. (2014) found that the use of cellulase and xylanase in wilted alfalfa (340 g DM/kg fresh crop) did not improve fermentation quality of silage. However, the addition of enzymes can result in a dose-dependent decrease of fiber (ADF, NDF) contents in silage according to dose (Nadeau et al., 2000; Khota et al., 2016). Other negative impacts were also found, such as excessive release of WSC (which may decrease aerobic stability), and in some cases the lower overall digestibility of consumed NDF by acting on the more-digestible components of NDF (Nadeau et al., 2000; Kung and Muck, 2015). Therefore, it is necessary to further study the application effect and mechanism of enzymes in silage.

Abundant metabolites are produced by microorganisms in silage. These metabolites have multiple effects in silage, such as improving fermentation quality and flavor, and prolonging aerobic stability. Organic acids produced by microorganisms in silage, such as conventionally detected acetic, formic, butyric, sorbic, isovaleric acids, play an important role in preventing the aerobic deterioration of silage (Ohyama and McDonald, 1975; Ohyama et al., 1977; Kung et al., 1998). Metabolomics shows that LAB in grass silage can produce more organic acids, such as 3-phenyllactic acid, 3-hydroxydecanoic acid, p-hydrocoumaric acid, hydroferulic acid, and p-coumaric acid (Broberg et al., 2007). In addition to organic acids, metabolomics allow to detect and quantify many other substances in silage. Actually, metabolomics analysis detected 280 metabolites and 102 different metabolites in LAB-treated alfalfa silage which include not only organic acids, but also amino acids and polyols (Guo et al., 2018). Some substances other than organic acids also have antibacterial activities, such as ethanol, acetaldehyde, 2, 3-butanedione, hydrogen peroxide, and exopolysaccharides (Driehuis and Elferink, 2000; Sjögren et al., 2003). At present, there are few metabolomics studies on silage, so it is necessary to further explore metabolites in silage.

The existing techniques, such as colony counting, terminal restriction fragment length polymorphism, denaturing gradient gel electrophoresis and real-time PCR, can only observe a limited number of species or the species with greater abundance in silage (Lin et al., 1992; Stevenson et al., 2006; Li and Nishino, 2011; Ridwan et al., 2015). In comparison, the next-generation sequencing (NGS) used in the present study can identify organisms with low abundance and then provide the composition of the whole microbial community in silage. Metabolomics targets all the small-molecular metabolites in the samples, and thus can reflect the changes in metabolite contents caused by different treatments, and as well as the correlation between the bacterial population and metabolites in silage (Xu et al., 2019). These new techniques could bring novel insights into silage research.

As mentioned above, unlike inoculants that directly promote LAB reproduction, cellulase promotes the growth of LAB by releasing water-soluble carbohydrates, but inoculants and cellulase both ultimately both improve the fermentation quality of silage by increasing the number of LAB. However, the exact compositions of the microflora and metabolites in silage may be different between inoculants-treated and enzyme-treated silages. Therefore, this study was conducted to determine the effect of *L. casei* treatment and cellulase treatment on (1) the fermentation quality of alfalfa silage; (2) the succession of bacterial community of alfalfa silage; (3) the ultra-microstructure of alfalfa silage; (4) the microbial metabolites of alfalfa silage.

## Materials and Methods

### Materials and Silage Preparation

Alfalfa was harvested at the first cut during the 30% bloom stage from fields located at the Inner Mongolia University for Nationalities (E122° 15′, N43° 38′), Inner Mongolia, in June 2018. The alfalfa was wilted to 289.2 g DM kg^–1^ in air ventilation indoor for 5 h, and then chopped to 10–20 mm lengths with a forage cutter. The crude protein (CP), neutral detergent fiber (NDF), acid detergent fiber (ADF), water-soluble carbohydrate (WSC), and ammonia nitrogen (NH3-N) in alfalfa forage were 206.2, 457.1, 336.2, 726.4, and 15.8 g kg^–1^, respectively. The pH of forage was 5.82 and LAB, molds, and yeast log10 cfu g^–1^ values were 6.06, 5.43, and 5.91, respectively. Treatments included (1) deionized water (CON); (2) cellulase (Main cellulase activities as: Endo-β-1,4-Glucanase (EC3.2.1.4): 20,000 IU g^–1^; Cellobiohydrolase (EC 3.2.1.91): 1,560 IU/g; β-Glucoside glycohydrolase (EC 3.2.1.21): 689 IU g^–1^; Endoxylanase (EC 3.2.1.8): 8,624 IU g^–1^) (SD-124, Challenge Co., Ltd., Beijing, China), applied at 5 g kg^–1^ of fresh forage (CE), and (3) *Lactobacillus casei* (BNCC189777, Bnbio Co., Ltd., Beijing, China), freshly cultured, applied at 1 × 10^6^ cfu g^–1^ of fresh forage (LC). The cellulase and the freshly-cultured inoculants were diluted with distilled water so that they were applied at the same rate (20 ml kg^–1^ FM); equal amounts of sterile distilled water were sprayed onto untreated forage. The alfalfa forage with given amount was sprayed with diluted additives (one sprayer for each treatment). Approximately 500 g of grass forage were vacuum sealed (Vacuum for 20 s, vacuum pressure 0.073MPa; DZ-260/PD, Afanlao Co., Ltd., Shanghai, China) in polyethylene silo bags (Size: 25 × 36 cm; Thickness: 0.24 mm) (Wangnuo Co., Ltd., Taizhou, China) (three silos per treatment). Silages were ensiled at room temperature (25–27°C) and sampled at 7–56 days. For air-exposed silage, the polyethylene bags containing 56 day silage were perforated (to formed 6 punctures evenly distributed on each side of silo bag) using a sterilized needle (3 mm in diameter) to allow air entry, placed in another opened polyethylene bag in the dark for 3 days (PO), and then sampled.

### Chemical and Microbial Analysis

The DM content of ensiled silage was determined after drying samples in a forced air oven at 65°C for 72 h. WSC content was measured according to Owens et al. (1999). Twenty-five grams of silage were diluted with 225 mL of de-ionized water, blended for 30 s, and filtered through four layers of gauze. The liquid fraction was measured for pH using an electrode pH meter (S20K, Mettler Toledo, Greifensee, Switzerland), and ammonia-N (NH_3_-N), which was analyzed as described by Zahiroddini et al. (2004). To measure organic acids, including lactic acid, acetic acid, propionic acid and butyric acid, filtrate was centrifuged at 1,800 × *g* for 15 min at 4°C and analyzed by high performance ion chromatography (ICS-3,000 system, Dionex, Sunnyvale, CA, United States) by conductivity detection. Organic acids were separated on an AS11 analytical column (250 mm × 4 mm) and an AG11 guard column under the following gradient conditions: potassium hydroxide; 0–5 min, 0.8–1.5 mM; 5–10 min, 1.5–2.5 mM, 10–15 min, 2.5 mM. The flow rate was 1.0 mL/min. For microbial counts, the filtrate (after gauze filtering) was subjected to serial dilutions and spread onto agar plates. The numbers of LAB were enumerated on spread plates of de Man, Rogosa, and Sharpe agar (Oxoid, CM 361). Yeasts and molds were enumerated on malt extract agar (Oxoid CM59) plates. Colonies were counted after incubation of the plates for 3 days at 30°C.

### Scanning Electron Microscopy Analysis

Leaves were sampled from fresh alfalfa and alfalfa silage ensiled for 56 days. The samples were fixed in 2.5% glutaraldehyde for at least 5 h, then were cleaned 3 times using 0.1M PBS. After this procedure, the samples were successively dehydrated with increasing ethanol concentrations (50, 70, 80, 90, 95, and 100% ethanol). Next, samples were cleaned in alcohol/isoamyl hexate (1:1) for 30 min followed by isoamyl hexate for 30 min. Then, samples were critical-point dried and sputtered with gold. Finally, the samples were visualized under a scanning electron microscope (Hitachi S-3,400, Tokyo, Janpan).

### Metabolite Profile Analysis

For silage extraction, 100 mg silage fodder samples from 3 days air exposure silages were added to 360 μL pre-chilled methanol and 40 μL internal standard (L-2-chlorophenylalanine, 0.3 mg/mL methanol). The mixture was homogenized, ultrasound treated, and centrifuged at 10,000 rpm at 4°C for 10 min; 400 μL of supernatant was then added to 80 μL methoxy amine hydrochloride (15 mg/ml in pyridine) for oximation. For derivatization, 80 μL BSTFA and 20 μL n-hexane were added to the oximation product and the mixture was placed at 70°C for 60 min then shaken for 2 min. The sample was then incubated at room temperature for 30 min. Gas chromatography-mass spectrometry (GC-MS) analysis was performed using an Agilent 7890A/5975C GC-MS instrument (Agilent, Santa Clara, CA, United States). Analytic compounds (1 μL) were injected in splitless mode with an inlet temperature of 260°C and separated with a HP-5MS (30 m × 0.25 mm × 0.25 μm) capillary column, using helium as a carrier gas at a constant flow rate (1 mL/min). The GC column temperature was increased from an initial temperature of 60–310°C at a rate of 8°C per minute and held for 6 min at 310°C. The ion sources temperature was 230°C and the quadrupole temperature was 270°C. Data acquisition was conducted using full scan mode with an m/z range of 50–600. GC-MS data were converted from ChemStation analysis files (version E.02.02.1431, Agilent, CA, United States) to netCDF format files, and were processed with Chroma TOF software (version 4.34, LECO, St Joseph, MI). Chroma TOF was used to extract raw peaks, filter and calibrate data baselines, and identify and integrate peaks. Metabolites were identified using Tracerfinder 3.2 (Thermo Fisher Scientific, CA) based on the NIST14.0 library. Majorbio Cloud Platform^1^ was used for multivariate analysis.

### Microbial Composition Analysis by Sequencing of 16S rRNA Gene

Genomic DNA was extracted from alfalfa samples using an E.Z.N.A.^®^ soil DNA Kit (Omega Bio-tek, Norcross, GA, United States.) according to the manufacturer’s instructions. The final DNA concentration and purification were determined using a NanoDrop 2,000 UV-vis spectrophotometer (Thermo Scientific, Wilmington, United States), and DNA quality was accessed by 1% agarose gel electrophoresis. The V3-V4 regions of bacterial 16S rRNA genes were amplified using 12 bps barcoded primers (forward 338F: 5′-ACTCCTACGGGAGGCAGCAG-3′; reverse 806R: 5′-GGACTACHVGGGTWTCTAAT-3′) by a thermocycler PCR system (GeneAmp 9,700, ABI, United States) (Yang et al., 2019). Each 20 μL reaction was composed of 4.0 μL of 5 × FastPfu Buffer, 2 μL of deoxynucleoside triphosphate (dNTP) mix (2.5 mM each), 0.4 μL of FastPfu DNA polymerase, 10 ng DNA, and 0.8 μL of each primer (5 μM). The PCR program for the thermocycler (ABI GeneAmp, United States) was: 95°C for 5 min to initial denaturation, then 27 cycles of 95°C for 30 s, 55°C for 30 s, and 72°C for 45 s, with a final extension of 72°C for 10 min. The PCR products were purified using an AxyPrep DNA Gel Extraction Kit (Axygen Biosciences, Union City, CA, United States), quantified using QuantiFluor^TM^-ST (Promega, United States), and cloned using an Ultra^TM^ DNA Library Prep Kit for Illumina (New England Biolabs Inc., Ipswich, MA, United States) as per the manufacturer’s instructions. Amplicons were pooled in equimolar amounts and paired-end sequenced (2 × 300) on an Illumina Miseq PE300 platform (Illumina Corporation, San Diego, United States) at the Shanghai Majorbio Bio-Pharm Technology Co., Ltd. (Shanghai, China).

### Bioinformatics Analysis

Raw Fastq files were merged by their overlapping regions with overlaps of > 10 bps using Trimmomatic (Bolger et al., 2014). The merged sequences were filtered, by removing sequences with a mismatch ratio up to 0.2 and bases containing N, to obtain optimized sequences. QIIME (Caporaso et al., 2010) was used to pick operational taxonomic units (OTUs) based on 97% sequence identity and to check sequence quality. Potential chimeras were removed using Usearch (version 7.1) (Edgar et al., 2011). OTUs present in negative control amplifications were also removed prior to analysis. Any OTU containing only 1 sequence was removed. Taxonomic assignments were produced by querying the representative sequences against the SILVA (Release132)^2^. Sequences belonging to chloroplast and mitochondria were removed from further analysis.

Alpha-diversity was obtained using Mothur 1.30 at the OTU level (Schloss et al., 2009). A cluster analysis of sample hierarchy was performed using the Qiime platform^3^ (Caporaso et al., 2010), Non-metric multidimensional scaling (NMDS) analysis was performed using Qiime and R vegan.

### Statistical Analysis

All microbial data was converted to log10 values. Chemical data are presented on a DM basis. Data on chemical determinations, microbial counts were subjected to two-way ANOVA using SPSS 19.0 (SPSS, Chicago, IL, United States), and the treatments and fermentation times were used as two fixed factors. When the interactions declared significant differences following the two-way ANOVA, one-way ANOVA was performed to evaluate the specific effect of treatments (CON, CE, LC) or ensiling time (ensiled for 7 days, ensiled for 56 days, and air exposure for 3 days) on the fermentation quality of alfalfa silage. Tukey’s HSD test was used to examine the differences between the various treatments (*P* < 0.05). All metabolite data were normalized using Simca-P software 11.5^4^ before hierarchical cluster analysis and principal component analysis (PCA). Significant differences in metabolites among treatment groups were tested by one-way analysis of variance. Quantitative normalization within replicates was transformed by a logarithmic base of 2 and MetaboAnalyst online analysis software^5^ was used to build a heatmap diagram. *P*-values below 0.05 between sample groups were considered significant. Metabolomic profiles from alfalfa silage analysis were processed using the Majorbio Cloud Platform^6^.

## Results

### Fermentation Characteristics of Alfalfa Silage After 56 Days of Ensiling

After 56 days of ensiling, the lowest pH was observed in the *L. casei* treated silage (LC) (4.60), followed by the cellulase treated silage (CE) (4.78) and the control silage (CON) (5.24) (Table 1 and Supplementary Table S1). The CE and LC had higher dry matter (DM) content than CON (*P* < 0.05). The levels of water soluble carbohydrates (WSC) were higher in CE and LC silages than in CON silage (*P* < 0.05). The ammonia level in LC remained lower than those of CON and CE on day 56 (*P* < 0.05).

**TABLE 1:** The fermentation characteristics of alfalfa silage during ensiling and air exposure.

Time (day)	Treatments	pH	DM (%)	WSC (g kg^–1^DM)	LA (g kg^–1^DM)	AA (g kg^–1^DM)	NH_3_-N (g kg^–1^DM)	LAB (log cfu g^–1^FM)	Mold (log cfu g^–1^FM)	Yeast (log cfu g^–1^FM)
7	CON	5.56a	28.25	38.12b	20.52b	2.56	1.70	7.30b	2.84	3.77
	CE	5.21b	28.70	45.24a	24.49b	2.97	1.56	8.35a	2.57	2.66
	LC	4.99b	28.55	34.00c	39.56a	2.59	1.71	8.65a	0	0
56	CON	5.24a	26.81b	14.89b	44.88b	10.59a	4.37a	8.37b	0	0
	CE	4.78b	27.53a	21.89a	58.68a	7.38b	3.86b	9.22a	0	0
	LC	4.60c	28.13a	18.77a	61.54a	5.88c	2.21c	9.44a	0	0
PO-3	CON	5.00a	27.27b	16.05b	25.06c	13.61b	3.71b	8.09c	3.81	3.72
	CE	4.87a	27.66ab	21.91a	32.80b	15.37a	4.52a	8.74b	3.28	3.61
	LC	4.64b	28.69a	18.22b	40.45a	8.46c	1.90b	9.11a	3.46	3.58
SEM		0.034	0.148	0.720	1.296	0.236	0.131	0.085	0.112	0.044
P-value	E	**	**	**	**	**	**	**	**	**
	T	**	**	**	**	**	**	**	**	**
	E × T	*	*	**	**	**	**	NS	**	**

DM, dry matter; FM, fresh matter; LA, lactic acid; AA, acetic acid; LAB, lactic acid bacteria. NH3-N, Ammonia-N; CON, untreated silage; CE, silages treated with cellulase; LC, silage treated with L. casei; PO-3, silages after air exposure of 3 days; SEM, error of the means; E, ensiling time; T, treatments; E × T, interaction of E and T; NS, P > 0.05; *0.001 < P < 0.01; **P < 0.001. Values within the same column with different letters are significantly different (P < 0.05).

After 56 days of ensiling, the CE and LC had more lactic acid and less acetic acid compared with CON (*P* < 0.05) (Table 1). CE and LC had greater LAB counts than CON (*P* < 0.05).

### Characteristics of Alfalfa Silage After Air Exposure

After the exposure to air, the pH in the LC was lower than in the CON and CE silages (*P* < 0.05) (Table 1). The DM content in LC was higher than in CON silages. The LC silage contained the greatest content of lactic acid (40.45 g kg^–1^), while CON silage had the lowest (25.06 g kg^–1^). The acetic acid content in CE was greatest and in LC was the lowest (*P* < 0.05). the LAB counts in LC was still the greatest (*P* < 0.05). The molds and yeasts in CON were not difference between groups (*P* > 0.05) (Table 1).

### The Effect of Time on the Fermentation Characteristics of Alfalfa Silage

During the ensiling, the pH decreased significantly in all silages and the fastest pH decrease occurred in LC silage (Table 1 and Supplementary Table S1). The dry matter DM contents in CON and CE silage significantly decreased (*P* < 0.05), but did not significantly change in LC silage (*P* > 0.05). The levels of WSC significantly decreased during ensiling in all silages (*P* < 0.05). The ammonia levels in CON and CE significantly increased during the ensiling (*P* < 0.05).

Both the lactic acid and acetic acid contents increased significantly in all silages (*P* < 0.05) during the ensiling (Table 1 and Supplementary Table S2). The number of LAB significantly increased in all silages during the ensiling (*P* < 0.05). Yeast and mold decreased to undetectable levels after 56 days of ensiling.

After the exposure to air, the pH in CON silage decreased significantly (*P* < 0.05), whereas it was not changed significantly in CE and LC silages (*P* > 0.05) (Table 1 and Supplementary Table S1). The DM and WSC of all groups did not vary significant (*P* > 0.05). The ammonia content of CON and LC did not change, but that of CE silage significantly increased (*P* < 0.05).

The lactic acid content significantly decreased after air exposure in all silages (*P* < 0.05) (Table 1 and Supplementary Table S2). The acetic acid levels in all silages increased significantly after air exposure (*P* < 0.05).

### Scanning Electron Microscopy (SEM)

**FIGURE 1:**
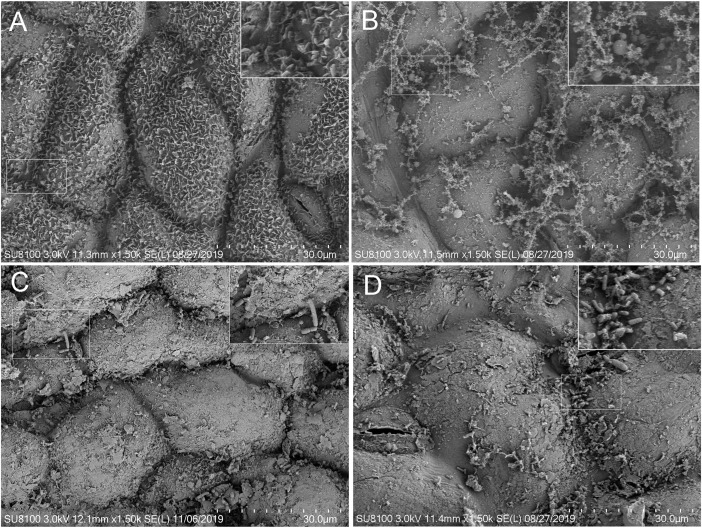
Scanning electron microscopy analysis (SEM) of the leaf of fresh and silage samples ensiled for 56 days with different treatments. **(A)** fresh alfalfa; **(B)** silage without treatment; **(C)** silage treated with cellulase; **(D)** silage inculated with *L. casei.*

SEM images revealed that the platelet structure of epicuticular wax crystals on the leaves of fresh alfalfa was intact (Figure 1A). Epicuticular wax appeared to be completely decomposed in CON silage (Figure 1B) and partly decomposed in CE and LC silages (Figures 1C,D). Furthermore, the images indicate that the leaves of CON and CE silages harbored both cocci and rods (Figures 1B,C); while the leaves of LC silage mainly harbored rods (Figure 1D).

### Diversity of the Bacterial Community

The rarefaction curves tended to plateau, and the average Good’s coverage of all samples was greater than 99%, suggesting that there was sufficient sequence depth to fully characterize the bacterial community diversity in silage samples (Table 2 and Supplementary Figure S1). The bacterial diversities among silage samples at day 56 were different according to Shannon-Weiner indexes (*P* < 0.05) (Supplementary Figure S2).

**TABLE 2:** Alpha-diversity of alfalfa silage during ensiling and air exposure for 3 days.

Treatments	Sobs	Shannon	Chao	Coverage
FA	370.25 ± 141.18	2.81 ± 0.38	402.80 ± 134.89	0.9914 ± 0.0032
CON7d	53.33 ± 11.01	1.76 ± 0.23	97.38 ± 38.76	0.9994 ± 0.0002
CE7d	68.75 ± 11.84	0.58 ± 0.30	123.79 ± 43.89	0.9993 ± 0.0001
LC7d	57.33 ± 2.08	0.52 ± 0.32	96.50 ± 12.77	0.9995 ± 0.0001
CON56d	65.6 ± 16.68	1.77 ± 0.09	158.56 ± 127.11	0.9995 ± 0.0002
CE56d	99.75 ± 42.27	1.15 ± 0.54	155.96 ± 46.43	0.9990 ± 0.0003
LC56d	60.5 ± 3.42	0.52 ± 0.17	126.03 ± 45.19	0.9991 ± 0.0001
CONPO	509.33 ± 311.36	2.27 ± 0.86	612.23 ± 318.94	0.9971 ± 0.0022
CEPO	93.25 ± 34.22	1.34 ± 0.32	185.57 ± 86.90	0.9990 ± 0.0003
LCPO	112 ± 30.05	1.00 ± 0.21	265.64 ± 87.77	0.9986 ± 0.0004

**FA, alfalfa before ensiling; CON, untreated silage; CE, silages treated with cellulase; LC, silage treated with L. casei; PO, silage from 3 days air exposure. 7 days, silage from 7 days ensiling; 56 days, silage from 56 days ensiling.**

**FIGURE 2:**
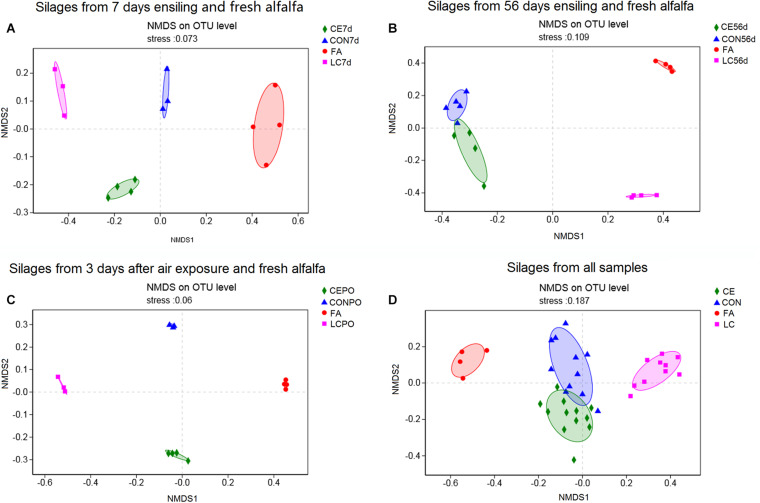
Non-metric multidimensional scaling analysis (NMDS) of bacterial community in fresh alfalfa silages with different treatements. **(A)** Silages from 7 days ensiling; **(B)**, silages from 56 days ensiling; **(C)**, silages from 3 days after air exposure; **(D)**, silages from all samples. FA alfalfa before ensiling; CON, Untreated silage; CE, silages treated with cellulase; LC, silage treated with *L. casei.*

NMDS analysis was used to reveal the distinct clusters among groups. For day 7 silages, CON, CE, LC, and fresh alfalfa (FA) distinctly clustered away from each other (Figure 2A) (Adonis based on bray curtis ditance, *R*^2^ = 0.86, *P* = 0.001), as were PO silages (Adonis based on bray curtis ditance, *R*^2^ = 0.81, *P* = 0.001) (Figure 2C). For day 56 silages, the FA, CON, CE, and LC distinctly clustered away from each other, but the clusters of CON and CE were close to each other (Adonis based on bray curtis ditance, *R*^2^ = 0.76, *P* = 0.001) (Figure 2B). On the whole, the silage samples from FA, CON, CE, and LC distinctly clustered away from each other, and CON and CE clusters were close to each other (Adonis based on bray curtis ditance, *R*^2^ = 0.58, *P* = 0.001) (Figure 2D).

### Bacterial Compositions of Alfalfa Silage

**FIGURE 3:**
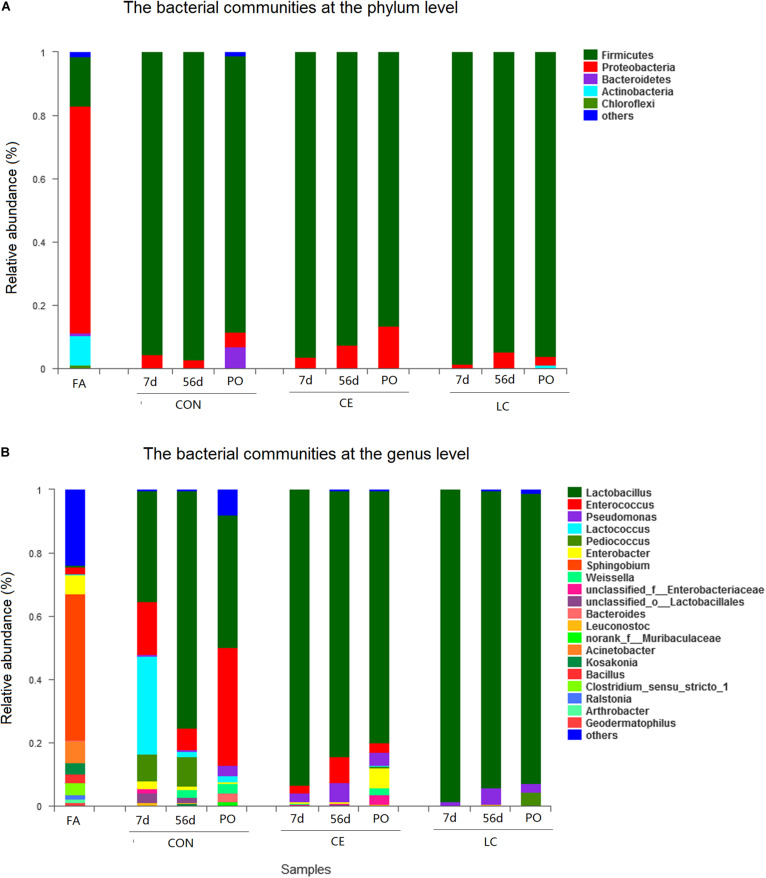
Bacterial composition of the alfalfa silage before and after ensilage with different treatments. Stacked bar represented percentage abundance. Small taxa with abundance below 0.01 were merged as others. FA, alfalfa before ensiling; CON, untreated silage; CE, silages treated with cellulase; LC, silage treated with *L. casei*, PO, silage from 3 days air exposure; 7 days, silage from 7 days ensiling; 56 days, silage from 56 days ensiling. The bacterial communities were showed at the phylum level **(A)**, the genus level **(B)**.

At the phylum level, proteobacteria dominated the bacterial communities in FA silage. Firmicutes dominated the bacterial communities in CON, CE, and LC silages (Figure 3A).

At the genus level, in fresh alfalfa, *Sphingobium* (46.26%) was dominant, followed by *Acinetobacter* (6.89%), *Enterobacter* (6.24), *Clostridium* (3.90%), Kosakonia (3.67%), *Enterococcus* (2.02%), and *Bacillus* (2.72%) (Figure 3B).

The dynamics of bacterial communities during ensiling was reflected profoundly at the genus level (Figure 3B).

After 7 days of ensiling, the CON silage mainly contained *Lactobacillus* (35.1%), *Lactococcus* (31.2%), *Enterococcus* (16.6%), and *Pediococcus* (8.3%). However, the CE and LC silages were dominated by *Lactobacillus* with relative abundance of 93.2 and 98.4%, respectively, which were higher in abundance than in CON (*P* < 0.05) (Figure 3B and Table 3).

**TABLE 3:** The relative abundance of *Lactobacillus* and *Enterococcus* during ensiling and air exposure (%).

		7 days	56 days	PO	SEM	*P*-value
*Lactobacillus*	CON	37.3 ± 16.44bB	75.10 ± 11.29bA	43.12 ± 10.39bB	6.58	**
	CE	93.19 ± 5.91a	83.93 ± 11.08ab	78.9 ± 12.87a	3.24	NS
	LC	98.37 ± 0.88a	94.17 ± 3.48a	91.20 ± 6.46a	1.42	NS
	P	**	*	**		
*Enterococcus*	CON	15.53 ± 9.15aB	6.41 ± 5.66B	36.17 ± 6.49aA	4.32	*
	CE	2.77 ± 4.17b	7.96 ± 9.87	3.02 ± 4.17b	1.88	NS
	LC	0.09 ± 0.12b	0.004 ± 0.005	0.001 ± 0.001b	0.02	NS
	SEM	2.66	1.90	5.42		
	P	*	NS	**		

P-values are shown as*0.01 < P ≤ 0.05,**0.001 < P < 0.01. CON, untreated silage; CE, silages treated with cellulase; LC, silage treated with L. casei; NS, no significant difference. a-b mean values within a column without a common letter differ; A-B means values within a row without a common letter differ.

After 56 days of ensiling, the relative abundance of *Lactobacillus* in CON had increased to 75.10%, followed by *Pediococcus* (9.3%) and *Enterococcus* (6.7%). *Lactobacillus* were still dominated in CE (83.93%) and LC (94.17%) silage and were significantly higher than CON (*P* < 0.05) (Table 3). *Enterococcus* (8.4%) and *Pseudomonas* (5.9%) in CE, and *Pseudomonas* (5.0%) in LC silages also presented (Figure 3B).

After 3 days of air exposure, the decrease in *Lactobacillus* (43.12%) and the increase of *Enterococcus* (36.17%) abundances were significant in CON silage (*P* < 0.05) (Figure 3B and Table 3). However, the abundance of *Lactobacillus* did not change significantly in both CE (79.7%) and LC (91.7%) silages, but these were still significantly greater than that of CON (*P* < 0.05) (Figure 3B and Table 3).

### Metabolomic Profiles of Alfalfa Silage After 3 Days of Air Exposure

**FIGURE 4:**
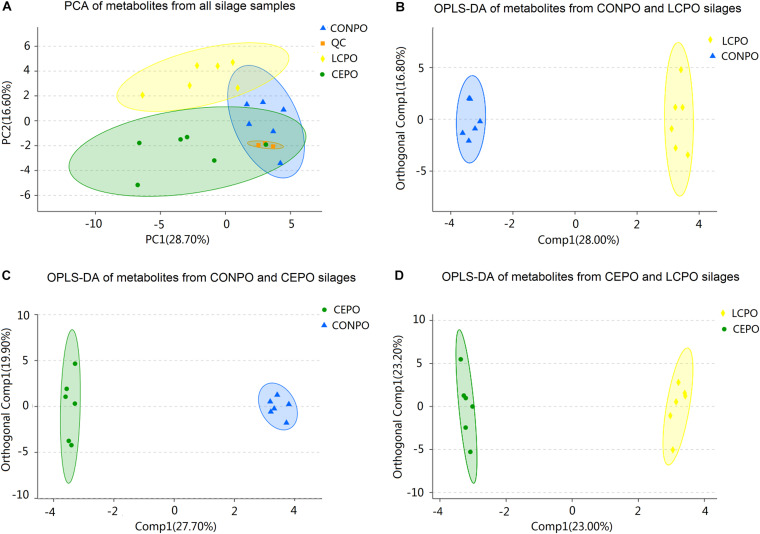
PCA and OPLS-DA scores plot of all metabolites features in air exposure alfalfa silages with different treatments. **(A)** The scores of the PCA model showing the directions that best explain the variance between the three treatements. **(B)** OPLS-DA scores plot of all metabolites features between the treatments of LCPO and CONPO. **(C)** OPLS-DA scores plot of all metabolites features between the treatment of CEPO and CONPO. **(D)** OPLS-DA scores plot of all metabolites features between the treatements of LCPO and CEPO. CONPO, untreated silage; CEPO, silages treated with cellulase; LCPO, silage treated with *L. paracasei*.

Based on the retention time and mass to charge ratio of total ions in the chromatograms of silage samples after 3 days of air exposure, a total of 196 metabolites were detected (Supplementary Table S3 and Supplementary Figure S3), and 95 different metabolites were identified (Table 4). Many metabolites were identified as sugars, organic acids, alcohols, ketones, and esters. According to PCA (Figure 4A), clear differences were revealed between treatments; The first axis (PC1) represented 28.7% of the variation among samples, and the second axis (PC2) explained 16.6% of the variation. Multivariate analysis OPLS-DA were applied to further reveal the differentiation silages, and the results showed a more obvious differentiation between the treatments (Figures 4B–D).

**TABLE 4:** Metabolites with significant differences between different treatments of silage alfalfa for 3 days of air exposure.

Metabolite	FCfCE/CON)	P_value	Metabolite	FC(LC/CON)	P_value	Metabolite	FC(LC/CE)	P_value
1-Indanone	1.44	*	Atropine	1.137	*	Ribose	1.102	*
5-Alpha-cholestan-3-one	1.13	**	Lactose	1.114	**	Aspartic acid	1.099	*
5 -Methylthioadenosine	1.102	*	Abietic acid	1.09	**	Trans,trans-muconic acid	1.079	**
Lactose	1.1	*	Lactamide	1.082	*	Abietic acid	1.069	*
Atropine	1.097	*	2-Hydroxybutanoic acid	1.078	***	4-Hydroxybutyrate	1.065	*
Lactamide	1.087	**	Gentisic acid	1.065	**	Hesperitin	1.063	*
2-Hydroxybutanoic acid	1.083	***	Trans,trans-muconic acid	1.063	*	Gentisic acid	1.057	*
Phytosphingosine	1.065	*	3,7,12-Trihydroxycoprostane	1.053	*	3,7,12-Trihydroxycoprostane	1.056	*
Mevalonic acid lactone	1 062	***	2,6-Diaminopimelic acid	1.047	**	Citraconic acid degr	1.054	***
Isoleucine	1.058	*	(+/-)-Taxifolin	1.044	**	Pantothenic acid	1.048	**
Carbazole	1.048	**	Cycloleucine	1.044	*	Gluconic lactone	1.047	*
Urocanicacid	1.048	**	6-Hydroxy caproic acid	1.039	***	(+/-)-Taxifolin	1.047	***
N-acetyl-L-leucine	0.962	**	Citraconic acid degr	1.03	**	Cycloleucine	1.046	**
Erythrose	0.962	**	5-Aminoimidazole-4-carboxamide	1.029	**	Beta-mannosylglycerate	1.043	*
2-Deoxy-D-glucose	956	*	Succinic acid	0.972	**	5,6-Dihydrouracil	1.038	**
Undine	0.955	**	N-acetyl-L-leucine	0.968	*	4-Hydroxyquinazoline	1.036	**
5beta-androstane-3,17-Dione	0.954	**	Purine riboside	0.961	*	L-Ciulonolactone	1.035	**
Benzyl thiocyanate	0.953	**	3-Hydroxypropionic acid	0.961	***	Lactic acid	1.021	***
Salicyl alcohol	0.952	*	Glyeolic acid	0.961	**	Succinie acid	0.969	**
Beta-mannosylgl ycerate	0.951	***	4-Hydroxymandelic acid	0.956	**	2-Hydroxybiphenyl	0.964	*
Alpha-toeopherol	0.95	**	Octanal	0.953	**	Carbazole	0.964	***
Androsterone	0.947	**	Citraconic acid	0.951	***	Uridine	0.961	*
Melibiose	0.944	*	3-(4-Hydroxyphenyl)propionic acid	0.948	**	Erythrose	0.961	**
Synephrine	0.944	*	Tryptophol	0.948	***	Lyxonic acid, 1,4-lactone	0.961	*
4-Hydroxybutyrate	0.942	*	Beta-hydroxypyruvate	0.947	**	6-Hydroxy caproic acid	0.955	***
Elesperitin	0.937	**	Fructose	0.947	**	2-Keto-isovaleric acid	0.954	**
Tryptophol	0.936	***	L-homoserine	0.944	**	3-(4-Hydroxyphenyl)propionic acid	0.95	**
3-Hydroxy L-proline	0.932	**	Lyxonic acid, 1,4-lactone	0.94	**	Urocanic acid	0.941	**
4-Vinyljihenol	0.93	**	21-Hydroxypregnenolone	0.939	**	Mevalonic acid lactone	0.941	***
Oxoproline	0.928	**	Lactulose	0.927	*	L-homoserine	0.934	**
N,N-dimethylarginine	0.927	*	Erythrose	0.925	***	Phytosphingosine	0.928	*
Purine riboside	0.925	***	Hydrocinnamic acid	0.923	**	Synephrine	0.92	**
Lactulose	0.918	*	Uridine	0.918	***	Hydrocinnamie acid	0.905	***
Phosphate	0.915	**	4-Vinylphenol	0.915	***	5-Alpha-cholestan-3-one	0.89	**
Epigallocatechin	0.913	*	Vanillylmandelic acid	0.898	***	Vanillylmandelic acid	0.886	***
Isopropyl-beta-D-Thiogalactopyranoside	0.91	*	Synephrine	0.869	***			
21-Hydroxypregnenolone	0.908	***	5,7-Dihydroxy-3-(4-methoxyphenyl)chromen-4-one	0.869	**			
Aspartic acid	0.908	**	1-Kestose	0.864	**			
Ribose	0.883	**						

FC, fold-changes; CON, untreated silage; CE, silages treated with cellulase; LC, silage treated with L. casei. “*” 0.01 < P < 0.05; “**” 0.001 < P < 0.01; “***” P < 0.001.

After 3 days of air exposure, 27 differential organic acids existed among the 56 identified organic acids (Tables 4, 5). The LC silage presented 6 organic acids more than CON silage, such as 2-hydroxybutanoic acid, citraconic acid, abietic acid, 6-hydroxy caproic acid, and gentisic acid. Urocanic acid, 2-hydroxybutanoic acid, 2-keto-isovaleric acid, and 6-hydroxy caproic acid dimer were higher in CE silage than in CON silage. Furthermore, 10 organic acids were found at higher concentrations in CON than in LC, such as benzoic acid, succinic acid, 3-hydroxypropionic acid, and 3-(4-hydroxyphenyl)propionic acid, and 7 organic acids were up-accumulated in CON than in CE, such as 3-hydroxypropionic acid, 4-hydroxybutyrate, aspartic acid, and beta-mannosylglycerate. Nine organic acids were more abundant in LC than in CE silage, such as abietic acid, 2, 3-dimethylsuccinic acid, gluconic acid, and lactic acid, 4-hydroxybutyrate, aspartic acid. Eight organic acids existed at higher concentrations in CE than in LC, such as, succinic acid, hydroxyphenyl)propionic acid, vanillylmandelic acid, and hydrocinnamic acid (Tables 4, 5).

**TABLE 5:** Metabolites belonging to sugars, amino acids, and organic acids in alfalfa silage treated with cellulase and *L. casei* after air exposure for 3 days.

	Fold-changes				Fold-changes		
Metabolite	LC/CON	LC/CE	CE/CON	Metabolite	LC/CON	LC/CE	CE/CON
**Sugars**				Citraconic acid	0.95***	0.99	0.96*
Lactose	1.11**	1.01	1.1*	Beta-hydroxypyruvate	0.95**	0.95	1
Fructose	0.95**	0.94	1	Hydrocinnamic acid	0.92**	0.91***	1.02
Lactulose	0.93*	1.01	0.92*	Vanillylmandelic acid	0.9**	0.89***	1.01
Erythrose	0.92***	0.96**	0.96**	2,3-Dimethylsuccinic acid	1.03	1.03*	0.99
1-Kestose	0.86**	0.94	0.91	Oxalic acid	1.02	1.01	1
Melezitose	1.03	1.06	0.97	Gluconic acid	1.02	1.03*	0.99
Raffinose	1.02	1.04	0.98	Pantothenic acid	1.02	1.05**	0.97
Levoglucosan	1	0.99	1.01	3,4-Dihydroxymandelic acid	1.02	1.03	1
Xylose	1	1.01	0.99	Gallic acid	1.01	1.01	0.99
3,6-Anhydro-D-galactose	0.99	1.02	0.97*	2-Ketocaproic acid	1.01	1.01	1
2-Deoxy-D-glucose	0.99	1.03	0.96*	Linolenic acid	1.01	1.02	0.99
d-Glucoheptose	0.99	1.01	0.98	Pelargonic acid	1.01	0.99	1.02
2-Deoxy-D-galactose	0.99	1.01	0.98	Quinic acid	1.01	1.03	0.99
Sophorose	0.99	0.99	1.01	4-Hydroxyphenylpyruvate	1.01	1.01	1
Leucrose	0.99	0.98	1.01	Fumaric acid	1.01	1.01	0.99
6-deoxy-D-glucose	0.98	0.98	1	Terephthalic acid	1.01	1.02	0.99
Melibiose	0.98	1.03	0.94*	Lactic acid	1	1.02***	0.98
Glucose	0.98	0.98	0.99	4-Hydroxybutyrate	1	1.07*	0.94*
Ribose	0.97	1.1*	0.88**	Aspartic acid	1	1.1*	0.91**
**Amino acid**				Acetylsalicylic Acid	1	0.98	1.02
Cycloleucine	1.04*	1.05**	1	D-Glyceric acid	1	1	1
L-homoserine	0.94**	0.93**	1.01	3-Hydroxybutyric acid	1	0.98	1.01
Oxoproline	0.95*	1.02	0.93**	Glucuronic acid	1	1.01	0.99
Isoleucine	1.06	1.03	0.97	Tartaric acid	1	1.01	0.99
Methionine	0.98	0.99	1.01	Threonic acid	0.99	1	0.99
N-Acetyl-L-leucine	0.96	0.97*	1.01	Beta-Mannosylglycerate	0.99	1.04*	0.95***
3-Hydroxy-L-proline	0.93	0.97	1.04*	Urocanic acid	0.99	0.94**	1.05**
**Organic acids**				Tartronic acid	0.99	0.97*	1.02
Abietic Acid	1.09**	1.07*	1.02	2-Keto-isovaleric acid	0.99	0.95**	1.03*
2-Hydroxybutanoic acid	1.08***	0.99	1.08***	2,2-Dimethylsuccinic Acid	0.99	1.02	0.97*
Gentisic acid	1.07**	1.06*	1.01	Galactonic acid	0.99	1.01	0.99
Trans,trans-Muconic acid	1.06*	1.08	0.98	L-Malic acid	0.99	0.99	0.99
2,6-Diaminopimelic acid	1.05**	1.03*	1.01	2-Hydroxyvaleric acid	0.99	1	0.98
6-Hydroxy caproic acid	1.04***	1.01	1.02	Lactobionic Acid	0.99	0.97	1.02
Benzoic acid	0.98*	0.98	0.99	2-Furoic Acid	0.98	1	0.98
Succinic acid	0.97**	0.97**	1	Saccharic acid	0.97	0.93	1.04
3-Hydroxypropionic acid	0.96***	0.98	0.98**	6-Hydroxynicotinic acid	0.96	0.98	0.97
Glycolic acid	0.96**	0.98*	0.98	3-Phenyllactic acid	0.96	1	0.97
4-Hydroxymandelic acid	0.96**	0.99	0.97**	6-hydroxy caproic acid dimer	0.99	0.95	1.03**
3-(4-Hydroxyphenyl)propionic acid	0.95**	0.95**	1				

*CON, untreated silage; CE, silages treated with cellulase; LC, silage treated with L. casei.*“*” 0.01 < *P* < 0.05; “**” 0.001 < *P* < 0.01; “***” *P* < 0.001.

Among the identified sugars, glucose levels were not different among the silages. Fructose, lactulose, erythrose, and 1-kestose were up-accumulated in CON than in LC, and lactulose, erythrose, 3, 6-anhydro-D-galactose, 2-deoxy-D-glucose, melibiose, glucose, and ribose levels were higher in the CON than in CE. However, only lactose level was higher in the treated silages (LC and CE) than in CON (Table 5).

Among the identified amino acids, cycloleucine concentration was greater in LC than in CON and CE. The 3-hydroxy-L-proline level was higher in CE than in CON. L-homoserine and oxoproline were more abundant in CON than in LC and oxoproline level were higher in CON than in CE (Table 5).

Lots of polyols, ketones and esters were identified in alfalfa silage (Supplementary Table S4). Many polyols in CON were up-accumulated than in LC and CE, such as acetol, salicyl alcohol, and tryptophol. Several ketones belonging to the hormone group were down-regulated in treated silages, such as 21-hydroxypregnenolone in LC and CE, and 5beta-androstane-3, 17-dione and androsterone in CE silage. The abundances of other substances, such as L-gulonolactone, 5-aminoimidazole-4-carboxamide, lactamide, and atropine in LC silage, and lactamide, mevalonic acid lactone, carbazole, atropine, and 1-indanone in CE silage, were also greater than those of control silage (Table 4 and Supplementary Figure S4).

### Correlation Between Metabolites and Bacterial Community

**FIGURE 5:**
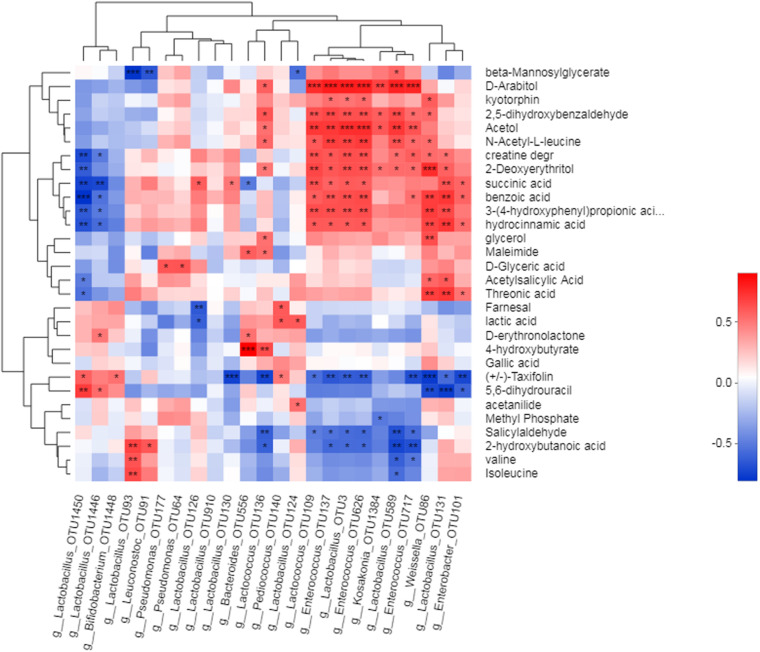
Correlation between metabolics and silage becteria. Heatmaps analyzed Spearman correlation coefficients (rho values) and *p*-values for pairwise comparisons of metabolites and bacterial genera. *P*-values are shown as *0.01 < *P* < 0.05, **0.001 < *P* < 0.01, ***P < 0.001. The clusters on the top and left of plot show the results of metabolites and hierarchical clustering of becterial taxa, respectively, based on Euclidean distance.

The correlation between metabolites and bacterial community was revealed by heat map analysis (Figure 5). Strong associations were revealed between a number of metabolites and silage bacteria, most of which were LAB. Among the 24 OTUs correlated with metabolites, 10 of them belong to *Lactobacillus*, and these OTU were mainly positively correlated with organic acids. For instance, OTU93 was positively correlated with 2-hydroxybutanoic acid, valine, and isoleucine. OTU126 and OTU130 were positively correlated with succinic acid. OTU124 positively was correlated with lactic acid. OTU3 was positively correlated with hydrocinnamic acid, 3-(4-hydroxyphenyl)propionic acid, benzoic acid, succinic acid, 2-deoxyerythritol, N-acetyl-L-leucine, acetol, 2, 5-dihydroxybenzaldehyde, and D-arabitol. OTU131 positively was correlated with threonic acid and acetylsalicylic acid. *Lactococcus* (OTU136) and *Bacteroides* (OTU556) positively correlated with 4-hydroxybutyrate. *Lactococcus* (OTU109), *Enterococcus* (OTU137, 626), and *Lactococcus* (OTU3) positively correlated with hydrocinnamic acid, 3-(4-hydroxyphenyl)propionic acid, benzoic acid, succinic acid, 2-deoxyerythritol, N-acetyl-L-leucine, 2, 5-dihydroxybenzaldehyde, and D-arabitol. *Weissella* (OTU86) and *Lactococcus* (OTU131) positively correlated with threonic acid, hydrocinnamic acid, 3-(4-hydroxyphenyl)propionic acid, benzoic acid, and 2-deoxyerythritol. *Kosakonia*, a non-LAB, was positively correlated with 2-deoxyerythritol, acetol, and D-arabitol.

## Discussion

Inoculants and enzymes are very important additives used in silage, especially for alfalfa, which is difficult to ferment to a lower pH and to obtain high quality silage from. In the present study, high-throughput sequencing and metabolomics revealed the dynamics of microbes during anaerobic and subsequent aerobic fermentation, and the characteristics of the metabolome of alfalfa silage treated with *L. casei* and cellulase. Cellulase is usually not a pure enzyme, but a synergistic multi-component enzyme system which hydrolyze cellulose into its monosaccharide, glucose (Woodward, 1991). In this study, the enzyme we used is mainly composed of endo-β-1, 4-glucanase, cellobiohydrolase, β-glucoside glycohydrolase, and endoxylanase. Actually, some other enzyme activities may be contained in commercial cellulase with small amounts, such as, xylanase, pectinase, glucoamylase, acetyl-xylanesterase, a-glucuronidase, β-D-xylosidase, α-D- arabinase, β-D glucosidase, and amylolytic enzyme (Khan et al., 1989; Spoelstra et al., 1992; Chen et al., 1994; Nadeau et al., 2000; Khota et al., 2016). SEM was used to visualize the changes on alfalfa leaf surfaces associated with different silage treatments.

### Fermentation Characteristics of Alfalfa Silage

Alfalfa was used with a relatively low dry matter content (29%) to improve the efficiency of enzymes. Tengerdy et al. (1991) reported that enzyme treatments were more effective in fresh (with DM content about 22%) than wilting alfalfa silage (with DM content about 35%). Previous studies showed the smaller effect of enzymes at higher DM content compared to the lower DM content, which may be because that the deprivation of water affects the distribution efficiency of enzyme (Van Vuuren et al., 1989; Kung et al., 1991; Sheperd and Kung, 1996; Su et al., 2019). Our study showed that the cellulase at the low DM content accelerated the fermentation of alfalfa and made the LAB enter the high abundance state more quickly.

After 56 days of ensiling, LC silage had greater lactic acid content, lower pH, and lower ammonia-N content than that of CON and CE. CE also showed resulted in a lower pH and ammonia-N content, and increased lactic acid production, than those of CON. In general, although both additives improved fermentation quality of alfalfa silage, *L. casei* was more effective than cellulase. Similar to our study, Tengerdy et al. (1991) and Su et al. (2019) also showed the improvement of fermentation quality in alfalfa silage added by LAB and enzymes.

After 3 days of air exposure, although the lactic acid content of all silages decreased greatly, the LC and CE maintained lower pH than that of CON silage. During the air exposure stage, the growth of yeast and mold was observed in silages. Yeast organic acid metabolism pathways are initiated by silage oxygenation, which can reduce lactic acid concentration and increase pH (Kung et al., 2018).

### Effect of *L. casei* and Cellulase on SEM Results of Alfalfa Silages

SEM analysis of alfalfa leaf surfaces ensiled for 56 days indicated that the treatment of alfalfa silage with cellulase and *L. casei* affected the decomposition of alfalfa leaves. The alfalfa cuticles are mainly composed of wax and cuticles, and epicuticular wax covers the surface of the cutin (Panciera and Krause, 1997). Our results showed that the platelet structure of epicuticular wax was intact on the leaf of fresh alfalfa. But on the leaf of CON, the epicuticular wax was completely decomposed, and cutin was exposed. However, on the leaves of LC and CE silage, the epicuticular wax and some platelet structures were preserved. The retention of the epicuticular wax may to a certain extent contribute to the high DM contents in the CE and LC. It was possible to observed on the SEM micrograph, that the leaves of CON silage harbored both cocci and rod-shaped bacteria, while LC silage dominantly harbored rod-shaped bacteria. The results of cocci and rods in the electron microscope view to some extent were linked to the bacterial communities of alfalfa silage determined by Illumina sequence, which showed that the rod-shaped bacterium *Lactobacillus* in LC was significantly more abundant than in CON, and there was certain abundance of cocci genera, such as *Enterococcus*, *Lactococcus*, and *Pediococcus* in CON silage.

### Effects of *L. casei* and Cellulase on Bacterial Community of Alfalfa Silage

Epiphytic bacterial communities in fresh forage are varying according to different environmental factors, such as geographical location, climate, and cutting (Langston and Bouma, 1960; Guo et al., 2018; Yang et al., 2019). In this study, several prevalent epiphytic bacteria were detected in fresh alfalfa, such as *Sphingobium*, *Acinetobacter*, *Enterobacter*, *Clostridium*, *Enterococcus*, and *Bacillus.* In contrast to our study, Yang et al. (2019) found that *Enterobacter* were the most abundant epiphytic bacteria in alfalfa silage, followed by *Pantoea*, unclassified_Cyanobacteria, and *Bacillus*. A study by Bao et al. (2016) revealed that *Bacillus megaterium*, *Enterobacter cloacae*, *Weissella cibaria*, *B. marisflavi*, *Pantoea agglomerans*, and *B. cereus* were epiphytes that existed in pre-ensiled alfalfa. In the present study, some epiphytic LAB species, such as *Lactobacillus* and *Enterococcus*, were found in a small amount in fresh alfalfa. After ensiling, although *Lactobacillus* became the predominant bacteria in silages, its abundance was lower in CON silage than in CE and LC silages. *Enterococcus* is cocci that initiate the early stage of ensiling, which are sensitive to low pH (Cai et al., 1998). In the present study, we found that *Enterococcus* were in high abundance in samples of CON silage from day 7 (16.63%) and 3 days of air exposure (37.21%). These results are in agreement with the *pH*-values of these silage samples, illustrating the effect pH and air exposure have on *Enterococcus* abundance. *Enterococcus* is a facultative anaerobic LAB, that can be isolated from silage. However, Cai (1999) used 5 *Enterococcus* strains as inoculants and found that *Enterococcus* did not improve silage quality. In this study, we found that *Enterococcus* was abundant in CON silage which showed a low level of ensiling quality.

Butyric acid-producing bacteria (BAB), such as Clostridia and *Bacillus*, are considered undesirable in silage (Muck, 2013). Both genera of bacteria can convert lactic acid into butyric acid, and some species of these genera can lead to the presence of pathogenic toxins in silage (Driehuis and Elferink, 2000). In this study, *Clostridium* and *Bacillus* were present in fresh alfalfa but were not detected during ensiling in all silages.

*Lactobacillus* were enriched in the ensiled silage and were extremely higher in abundance in treated (LC and CE) silage compared to untreated silage. *Lactobacillus* can ferment hexose to produce lactic acid in homofermentative species or ferment hexose and pentose to produce equal amounts of lactic acid and acetic acid in heterofermentative species (Parvin and Nishino, 2009; Muck et al., 2018). In terms of the silage quality in this study, the dominant of *Lactobacillus* were responsible for the higher lactic acid and acetic acid content, the lower pH in the LC and CE silages, compared to the control silage.

Lots of research shows the domination of *Lactobacillus* in the ensiled silages, including those of alfalfa (McGarvey et al., 2013; Zheng et al., 2017), corn (Li and Nishino, 2011; Ogunade et al., 2017), guinea grass (Parvin and Nishino, 2009), and *Calliandra calothyrsus* and *Pennisetum purpureum* (Ridwan et al., 2015). Our research was consistent with previous research, revealing the prevalence of *Lactobacillus* in inoculated alfalfa silages, with high abundance in LC silage and low abundance (from 10% to approximately 30%) in CON silage (McGarvey et al., 2013; Zheng et al., 2017).

Air exposure (PO) affected the bacterial composition of the control and treated silages in different ways. Compared to the ensiling silages, air exposure decreased the abundance of *Lactobacillus* and the increased abundance of *Enterococcus* in CON silage, while it did not apparently decrease *Lactobacillus* abundance in CE and LC silages. On the contrary, the pH of CON decreased after aerobic exposure, while the pH of the treated silages (LC and CE) increased, which was caused by the increase of *Enterococcus* in CON (Tables 1–4). As mentioned above, *Enterococcus* can generate organic acids. There is a lack of research on bacterial community during aerobic exposure. McGarvey et al. (2013) ever reported the change of bacterial population structure of alfalfa silage during air exposure, with increases in Firmicutes, Actinobacteria, and Fusobacteria abundance and a decrease in Proteobacteria. Although LAB still dominated air-exposed silage, the facultative and obligate heterofermentative species of them can produce acetic acid in addition to lactic acid (Driehuis and Elferink, 2000; Muck et al., 2018). Acetic acid can improve the aerobic stability of silage by inhibiting fungal growth (Wilkinson and Davies, 2013; Kung et al., 2018).

Cellulase can hydrolyze cellulose fibers to soluble carbohydrates which are substrates of LAB growth. We found that cellulase vigorously boosted the growth of *Lactobacillus* in alfalfa silage. The abundance of *Lactobacillus* in day 7 silage indicates that the implication of *Lactobacillus* in CE is almost as fast as that of LC silage, indicating the high effective of cellulase in silage. This may be related to high moisture of silage which facilitates the activity of enzyme (Van Vuuren et al., 1989; Sheperd and Kung, 1996). The final abundance of *Lactobacillus* in CE silage (up to 84.51%) in our study was greater than that in a recent study by Su et al. (2019), which showed that only 22.91% *Lactobacillus* existed in CE alfalfa silage ensiled for 60 days. This is because that the dose of the enzyme in our study (20,000IU, 0.5% of fresh matter) was higher than in the study of Su et al. (2019) (10,000 U/g; 0.1% of fresh matter). The higher enzyme activity released more sugars which promotes the growth of LAB. In our study, bacterial communities at OTU level showed that the OTU belonging to *Lactobacillus* in CE silage was not the same as that of LC silage. This indicates that different LAB strains developed in different treatment conditions.

### Effects of *L. casei* and Cellulase on Metabolomic Profiles in Alfalfa Silage

Metabolomic profile analysis revealed that the treatments of alfalfa silage with cellulase or *L. casei* modulated the metabolite composition pattern differently after air exposure. A total of 196 metabolites were detected, and 95 metabolites showed difference in relative concentrations between conditions, and most of these metabolites were organic acids, sugars, polyols, and ketones. A total of 280 metabolites were found in the previous investigation of the metabolomic profile in alfalfa silage ensiled for 90 days (control and *L. plantarum* and *L. buchneri*-inoculated groups), and 102 substances with altered concentrations by different treatments (Guo et al., 2018). Some of the differentially concentrated substances or their analog were identified in our study, including aspartic acid, lactic acid, glycerol, succinic acid, benzoic acid, adenine, arabitol, and mannitol. The treatment of different additives and air penetration may have been the main reasons for the differences in metabolites and their content between their study and our study.

Organic acids can improve the aerobic stability of silage (Ohyama and McDonald, 1975; Ohyama et al., 1977; Sjögren et al., 2003; Broberg et al., 2007). Furthermore, organic acids all have sourness, and some have characteristic flavor, taste or aroma (Whiting, 1976). Some studies have used metabolomics techniques to explore metabolites in silage, and have found many organic acids (Guo et al., 2018; Xu et al., 2019). However, the metabolites revealed in these studies were formed happened in the ensiling stage and did not reflect the situation in aerobic exposure. Our study found that 57 organic acids were produced during the aerobic exposure of alfalfa silage. Among them, 2-hydroxybutanoic acid, 3-hydroxypropionic acid, 3-hydroxybutyric acid, 4-hydroxybutyrate, saccharic acid, 3-phenyllactic acid, aspartic acid, benzoic acid, D-glyceric acid, hydrocinnamic acid, glycolic acid, glucuronic acid, linolenic acid, L-malic acid, and threonic acid were reported before (Broberg et al., 2007; Guo et al., 2018; Xu et al., 2019). Compared to control silage, both the treatment of *L. casei* and cellulase resulted in an up-accumulation of organic acids. Compared to CE silage, *L. casei* treatment resulted in up-regulation of more organic acid, especially lactic acids. However, different from Guo et al. (2018), the CON in our study also showed more abundant organic acids than LC or CE, which may be because air exposure caused more complex conditions, such as increased microbial diversity and fungi growth.

Sugars are an important nutrients for microbial growth. In the identified sugars, only lactose level was higher in LC and CE than in CON, and the levels of most sugars in the LC and CE were lower than those in CON. Lactose is a disaccharide and is made up of glucose and galactose. Lactose may be produced by the decomposition of glycosides in alfalfa under the action of microorganisms. The main glycosides in alfalfa are raffinose, stachyose, and saponin (Cunningham et al., 2003). Saponin highly exist in alfalfa and is made up of glucose, galactose, rhamnose, arabinose, xylose, and other pentose sugars (Tava and Odoardi, 1996; Pecetti et al., 2010; Rafińska et al., 2017). Besides lactose, galactose, xylose, raffinose, and fructose were identified in our study. Lactose is an important fermentation substrate for LAB but not for many pathogenic and spoilage organisms, so the presence of lactose can help to decrease the pH of silage and inhibit the growth of spoilage organisms (Vos and Vaughan, 2010). Glucose level was lower compared with other sugars and not significantly different among the groups, suggesting that the effect of glucose on bacterial flora was reduced during aerobic exposure.

Lots of polyols, ketones and esters were identified in alfalfa silage. Many polyols in the control silage were up-accumulated than LC and CE silage. This may be caused by the higher diversity of bacterial community and the increase of spoilage microorganism in the control silage compared to the treated silage. Several ketones belonging to the hormone group were down-regulated in the treated silages. This may help to reduce the effects of hormones on animals, if animals eat the silage after 3-days of aerobic exposure. Noticeably, only 7 amino acid species were detected in alfalfa silage after aerobic exposure, which was far less than that of alfalfa silage (14 amino acids) in the study of Guo et al. (2018), indicating that many amino acids were decomposed due to aerobic exposure. The role of some metabolites, especially the metabolites that different between groups, in the bacterial community and silage quality need for more research during air exposure.

### Correlations Between Bacterial Community and Metabolites of Alfalfa Silage

Most metabolites, mainly organic acids and a few polyols or aldehydes, were positive to the growth of LAB such as *Lactobacillus*, *Lactococcus*, and *Enterococcus*. The correlation analysis between metabolites and bacterial community revealed that 24 OTUs were correlated with metabolites, and 10 of them belong to *Lactobacillus*. The metabolites which were positively associated with OTU of *Lactobacillus* contained 8 organic acids, 3 amino acids, and 3 polyols. These organic acids and polyols contributed to the aerobic stability of silages. As a dominant bacterial genus, *Lactobacillus* may to some extent prevent aerobic spoilage and maintain feed quality during the initial stage of aerobic exposure. *Lactococcus*, *lactococcus*, *Weissella*, and *Enterococcus* were also positively associated with lots of organic acids. This indicates a positive role of LAB on increasing the aerobic stability during the air penetration period by the secretion of metabolites. Furthermore, aerobic enterobacterial *Kosakonia* was found to positively correlated with 2-deoxyerythritol, acetol, and D-arabitol, and these sugar-alcohols usually have a sweet or spicy taste.

## Conclusion

High-throughput sequencing confirmed the hypothesis that the improvement in the quality of inoculated silage is related to its bacterial community. Both *L. casei* and cellulase greatly increased the abundance of *Lactobacillus* in alfalfa silage, which corresponded with good fermentation quality. Quality indicators of high levels of lactic and acetic acids, and low pH both in the ensiling and after air exposure were found in *L. casei* and cellulase treated samples. Air exposure influenced the bacterial composition of control silage, which showed decreased *Lactobacillus* and increases *Enterococcus* abundance. The bacterial composition of CE and LC silages were slightly influenced by air exposure, with no decrease in *Lactobacillus*. Scanning electron microscopy analyses revealed that *L. casei* led to more preserved epicuticular wax crystals on the leaf of alfalfa silage. The results revealed that *L. casei* can readily colonize and dominate alfalfa silage and lead to a higher fermentation quality over the ensiling time and after air exposure than control and cellulase treated silage. Metabolomic profiling analysis revealed more organic acids were produced in *L. casei* and cellulase treated silage after air exposure for 3 days. Based on our study, *L. casei* is recommended as an inoculant for alfalfa ensiling. Many metabolites were revealed by metabolomic profiling during air exposure; further research is required to determine their roles in the ensiling process and in silage quality.

## Data Availability Statement

The datasets generated for this study can be found in the NCBI BioProject ID PRJNA595713.

## Author Contributions

ZH and DM conceived and designed the experiments. ZH, HN, JY, JC, SL, and SZ performed the experiments. ZH, HN, and QT analyzed the data and contributed reagents, materials, analysis tools prepared figures and tables. DM and ZH authored and reviewed drafts of the manuscript, approved the final draft. All authors contributed to the article and approved the submitted version.

## Conflict of Interest

The authors declare that the research was conducted in the absence of any commercial or financial relationships that could be construed as a potential conflict of interest.

## Footnotes

1 https://cloud.majorbio.com

2 http://www.arb-silva.de

3 http://qiime.org/scripts/assign_taxonomy.html

4 http://www.umetrics.com/simca

5 www.metaboanalyst.ca/

6 www.majorbio.com
