# Supplementary material for: The Microbiota Dynamics of Alfalfa Silage During Ensiling and After Air Exposure, and the Metabolomics After Air Exposure Are Affected by Lactobacillus casei and Cellulase Addition
Source: Front Microbiol. 2020 Nov 26;11:519121. doi: 10.3389/fmicb.2020.519121 (PMC7732661; doi:10.3389/fmicb.2020.519121)
Supplement: Supplementary file 1 [file Table_1.DOCX]

Table S1 Fermentation characteristics of alfalfa silage during ensiling and air exposure

| Items | treatments | ensiling time | |  | |  |  | |  | |  |
| --- | --- | --- | --- | --- | --- | --- | --- | --- | --- | --- | --- |
|  |  | 7d | 56d | | PO | | | SEM | | P-value | E×T |
| pH | CON | 5.56±0.07aA | 5.24±0.06aB | | 5.00±0.04aC | | | 0.08 | | *** |  |
|  | CE | 5.21±0.04bA | 4.78±0.05bB | | 4.87±0.09aB | | | 0.07 | | *** | ** |
|  | LC | 4.99±0.03cA | 4.60±0.04cB | | 4.64±0.08bB | | | 0.06 | | *** |  |
|  | SEM | 0.08 | 0.10 | | 0.06 | | |  | |  |  |
|  | P-value | ** | *** | | ** | | |  | |  |  |
| DM (%) | CON | 28.25±0.12A | 26.81±0.10bC | | 27.27±0.22bB | | | 0.22 | | *** |  |
|  | CE | 28.70±0.16A | 27.53±0.31aB | | 27.66±0.34abB | | | 0.20 | | ** | ** |
|  | LC | 28.55±0.20 | 28.13±0.28a | | 28.69±0.39a | | | 0.12 | | NS |  |
|  | SEM | 0.08 | 0.20 | | 0.23 | | |  | |  |  |
|  | P-value | * | * | | ** | | |  | |  |  |
| WSC | CON | 38.12±1.24bA | 14.89±0.97bB | | 16.05±0.70bB | | | 3.79 | | *** |  |
| (g kg^-1^ DM) | CE | 45.24±1.64aA | 21.89±1.69aB | | 21.91±1.12aB | | | 3.91 | | *** | *** |
|  | LC | 34.00±1.43cA | 18.77±1.14aB | | 18.22±0.92bB | | | 2.61 | | *** |  |
|  | SEM | 1.69 | 1.08 | | 0.90 | | |  | |  |  |
|  | P-value | *** | ** | | ** | | |  | |  |  |
| Ammonia-N | CON | 1.70±0.18C | 4.37±0.23aA | | 3.71±0.32bB | | | 0.41 | | *** |  |
| (g kg^-1^ DM) | CE | 1.56±0.11C | 3.86±0.14bB | | 4.52±0.13aA | | | 0.45 | | *** | *** |
|  | LC | 1.71±0.31 | 2.21±0.16c | | 1.90±0.32c | | | 0.11 | | NS |  |
|  | SEM | 0.07 | 0.33 | | 0.39 | | |  | |  |  |
|  | P-value | NS | *** | | *** | | |  | |  |  |

CON, untreated silage; CE, silages treated with cellulase; LC, silage treated with *L. casei*; E, ensiling time; T, treatment; DM, dry matter; WSC, water resolved carbohydrate.

“*” 0.01<P <0.05; “**” 0.001< P < 0.01; “***” P < 0.001
