# Supplementary material for: The Microbiota Dynamics of Alfalfa Silage During Ensiling and After Air Exposure, and the Metabolomics After Air Exposure Are Affected by Lactobacillus casei and Cellulase Addition
Source: Front Microbiol. 2020 Nov 26;11:519121. doi: 10.3389/fmicb.2020.519121 (PMC7732661; doi:10.3389/fmicb.2020.519121)
Supplement: Supplementary file 2 [file Table_2.DOCX]

Table S2 Fermentation characteristics of alfalfa silage during ensiling and air exposure

|  | Treatments |  | ensiling time |  |  |  |  |
| --- | --- | --- | --- | --- | --- | --- | --- |
|  |  | 7d | 56d | PO | SEM | P-value | E×T |
| Lactic acid g kg^-1^ | CON | 20.52±1.16bB | 44.88±0.67bA | 25.06±0.87cB | 3.79 | *** |  |
|  | CE | 24.49±0.64bC | 58.68±1.13aA | 32.80±1.14bB | 5.39 | *** | *** |
|  | LC | 39.56±1.49aC | 61.54±1.33aA | 40.45±1.07aC | 4.41 | *** |  |
|  | SEM | 3.38 | 5.35 | 4.00 |  |  |  |
|  | P-value | *** | *** | ** |  |  |  |
| Acetic acid g kg^-1^ | CON | 2.56±0.66C | 10.59±0.73aB | 13.61±0.48bA | 1.66 | *** |  |
|  | CE | 2.97±0.47C | 7.38±0.38bB | 15.37±0.56aA | 1.82 | *** | *** |
|  | LC | 2.59±0.89C | 5.88±0.18cB | 8.46±0.29cA | 0.85 | *** |  |
|  | SEM | 0.09 | 0.71 | 1.04 |  |  |  |
|  | P-value | NS | *** | *** |  |  |  |
| LAB,log10 cfu g^-1^ | CON | 7.3±0.24bB | 8.37±0.09bA | 8.09±0.15cA | 0.17 | ** |  |
|  | CE | 8.35±0.21aC | 9.22±0.11aA | 8.74±0.07bB | 0.13 | ** | NS |
|  | LC | 8.65±0.15aC | 9.44±0.09aA | 9.11±0.12aB | 0.12 | ** |  |
|  | SEM | 0.21 | 0.17 | 0.15 |  |  |  |
|  | P-value | *** | *** | *** |  |  |  |
| Mould,log10 cfu g^-1^ | CON | 2.84±0.29B | <2.0 | 3.81±0.29A |  |  |  |
|  | CE | 2.57±0.19 | <2.0 | 3.28±0.14 |  |  |  |
|  | LC | <2.0 | <2.0 | 3.46±0.34 |  |  |  |
|  | SEM |  |  | 0.11 |  |  |  |
|  | P-value |  |  | NS |  |  |  |
| Yeast,log10 cfu g^-1^ | CON | 3.77±0.10aA | <2.0 | 3.72±0.07A |  |  |  |
|  | CE | 2.66±0.11bB | <2.0 | 3.61±0.10A |  |  |  |
|  | LC | <2.0 | <2.0 | 3.58±0.12 |  |  |  |
|  | SEM |  |  | 0.08 |  |  |  |
|  | P-value |  |  | NS |  |  |  |

CON, untreated silage; CE, silages treated with cellulase; LC, silage treated with *L. casei*; NS, no significant difference.

“*” 0.01<P <0.05; “**” 0.001< P < 0.01; “***” P < 0.001
