# Supplementary material for: The Microbiota Dynamics of Alfalfa Silage During Ensiling and After Air Exposure, and the Metabolomics After Air Exposure Are Affected by Lactobacillus casei and Cellulase Addition
Source: Front Microbiol. 2020 Nov 26;11:519121. doi: 10.3389/fmicb.2020.519121 (PMC7732661; doi:10.3389/fmicb.2020.519121)
Supplement: Supplementary file 5 [file Table_5.docx]

| **Metabolite** | **LC/CON** | **LC/CE** | **CE/CON** | **Metabolite** | **LC/CON** | **LC/CE** | **CE/CON** |
| --- | --- | --- | --- | --- | --- | --- | --- |
| **Esters** |  |  |  | 5Beta-androstane-3,17-dione | 0.96 | 1 | 0.95** |
| L-Gulonolactone | 1.03* | 1.04** | 1 | Androsterone | 0.96 | 1.01 | 0.95** |
| Lyxonic acid, 1,4-lactone | 0.94*** | 0.96* | 0.98 | 1-Indanone | 1.24 | 0.86 | 1.44* |
| Gluconic lactone | 1.03 | 1.05* | 0.98 | **Polyols** |  |  |  |
| Ethyl cinnamate | 1.02 | 1.02 | 1 | Acetol | 0.98** | 1 | 0.97** |
| Mono(2-ethylhexyl)phthalate | 1.02 | 1.02 | 1 | Benzyl alcohol | 0.98* | 0.99 | 0.99 |
| D-erythronolactone | 1.02 | 1.02** | 0.99 | Salicyl alcohol | 0.97* | 1.02 | 0.95* |
| Linoleic acid methyl ester | 1.01 | 1.02* | 0.98 | Tryptophol | 0.95*** | 1.01 | 0.94*** |
| Methyl octadecanoate | 1.01 | 1.02 | 0.99 | Piceatannol | 1.02 | 1.02 | 1 |
| Docosanoic acid methyl ester | 1.01 | 1.02 | 0.99 | Glycerol | 1 | 1.01 | 0.99 |
| Methyl icosanoate | 1.01 | 1.02 | 0.99 | 4-Hydroxy phenylethanol | 1 | 1 | 0.99 |
| Methyl hexadecanoate | 1 | 1.01 | 0.99 | Threitol | 1 | 1.02 | 0.98 |
| Methyl decanoate | 1 | 1.01 | 0.99 | Farnesol | 1 | 1.01 | 1 |
| Tetradecanoic acid, methyl ester | 1 | 1.01 | 0.99 | 3-Methylamino-1,2-propanediol | 1 | 1 | 0.99 |
| Tetracosanoic acid, methyl ester | 1 | 1.02 | 0.99 | Coniferyl alcohol | 1 | 1.01 | 0.99 |
| Methyl dodecanoate | 1 | 1.02 | 0.99 | (+-)-Dihydrocarveol | 1 | 1.01 | 0.99 |
| Mevalonic acid lactone | 1 | 0.94*** | 1.06*** | 4-Hydroxy-3-methoxybenzyl alcohol | 1 | 1.01 | 0.99 |
| Methyl Palmitoleate | 0.99 | 1 | 0.99 | Ribitol | 1 | 0.99 | 1.01 |
| Benzyl thiocyanate | 0.99 | 1.03 | 0.95** | Phytol | 1 | 1.01 | 0.99 |
| **Ketones** |  |  |  | Xylitol | 0.99 | 0.99 | 1 |
| Dihydroxyacetone | 0.98*** | 0.99 | 0.99 | Diglycerol | 0.99 | 1 | 1 |
| 21-hydroxypregnenolone | 0.94** | 1.03 | 0.91** | Mannitol | 0.99 | 0.99 | 1 |
| 3-Hydroxyflavone | 1.02 | 1.02*** | 0.99 | D-Arabitol | 0.98 | 1.01 | 0.97** |
| Adrenosterone | 0.99 | 1.15 | 0.86 | 4-Methyl-5-thiazolethanol | 0.96 | 0.97 | 0.98 |
| Estrone | 0.98 | 0.99 | 0.99 | Sorbitol | 0.95 | 0.94 | 1.01 |

**Table S5** Metabolites belonging to esters, ketones, and polyols in alfalfa silage treated with cellulase and *L. casei* after air exposure for 3 days

CON, untreated silage; CE, silages treated with cellulase; LC, silage treated with *L. casei*;

“*” 0.01<P <0.05; “**” 0.001< P < 0.01; “***” P < 0.001
