# Supplementary figures and images for: The Microbiota Dynamics of Alfalfa Silage During Ensiling and After Air Exposure, and the Metabolomics After Air Exposure Are Affected by Lactobacillus casei and Cellulase Addition
Source: Front Microbiol. 2020 Nov 26;11:519121. doi: 10.3389/fmicb.2020.519121 (PMC7732661; doi:10.3389/fmicb.2020.519121)

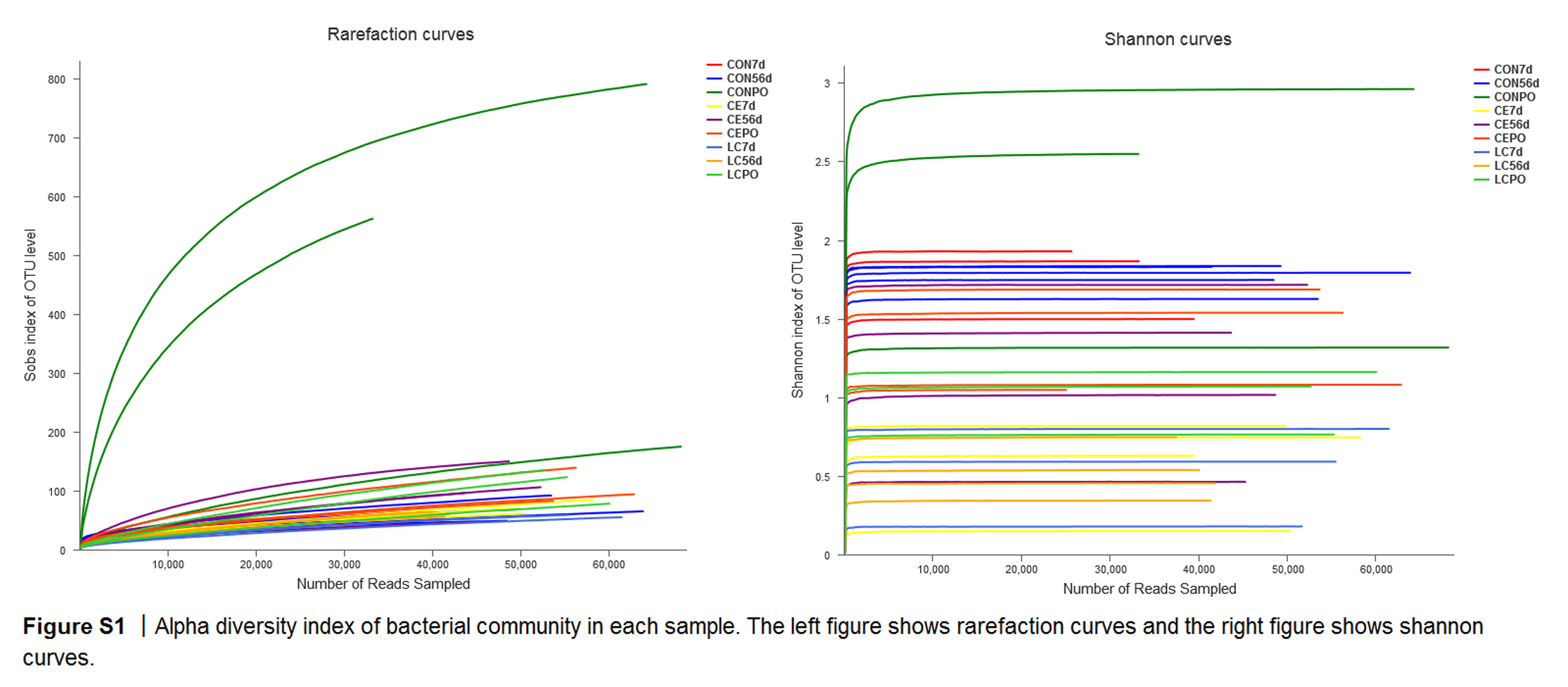

Supplement: Supplementary file 6 [file Image_1.TIF]

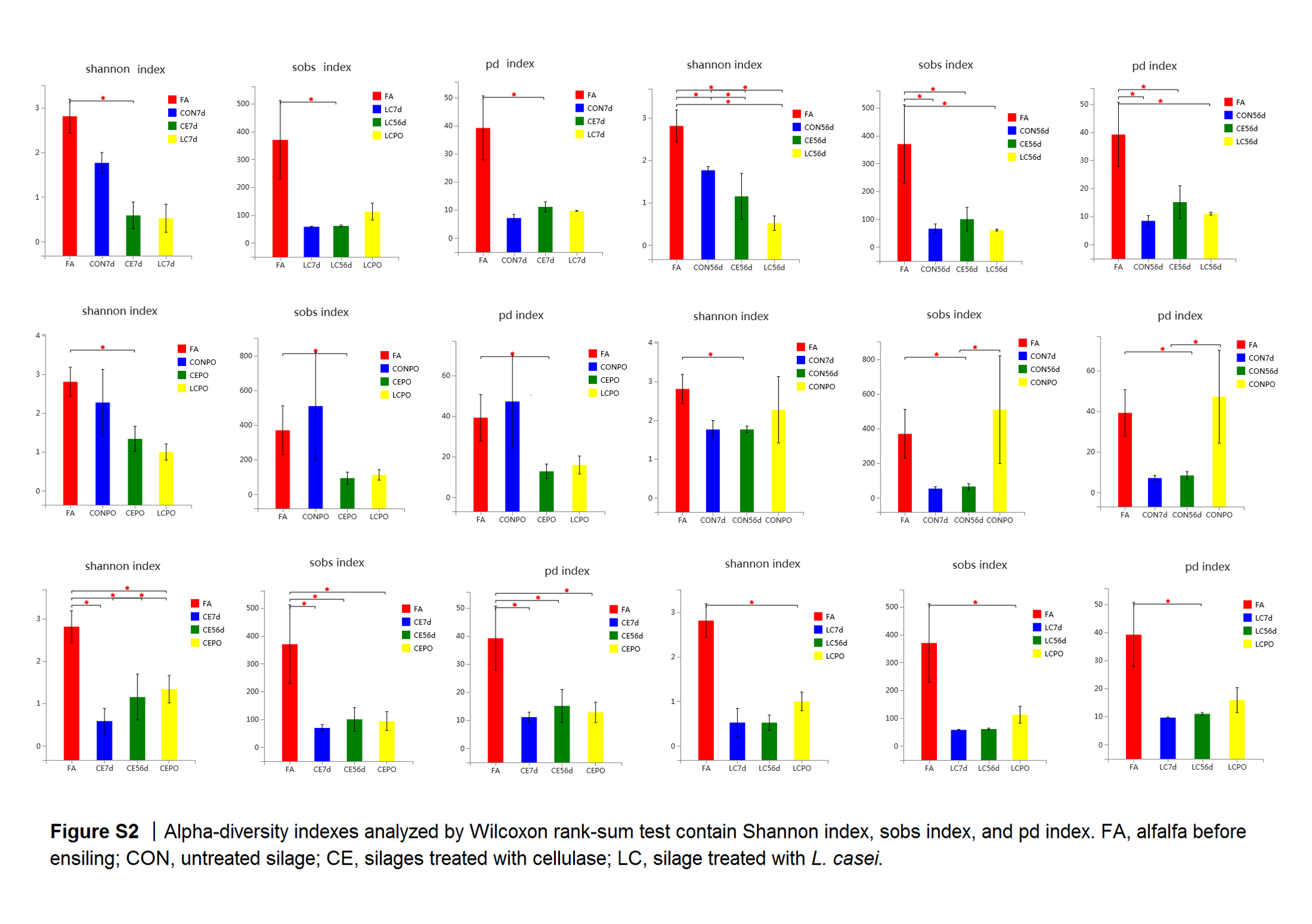

Supplement: Supplementary file 7 [file Image_2.TIF]

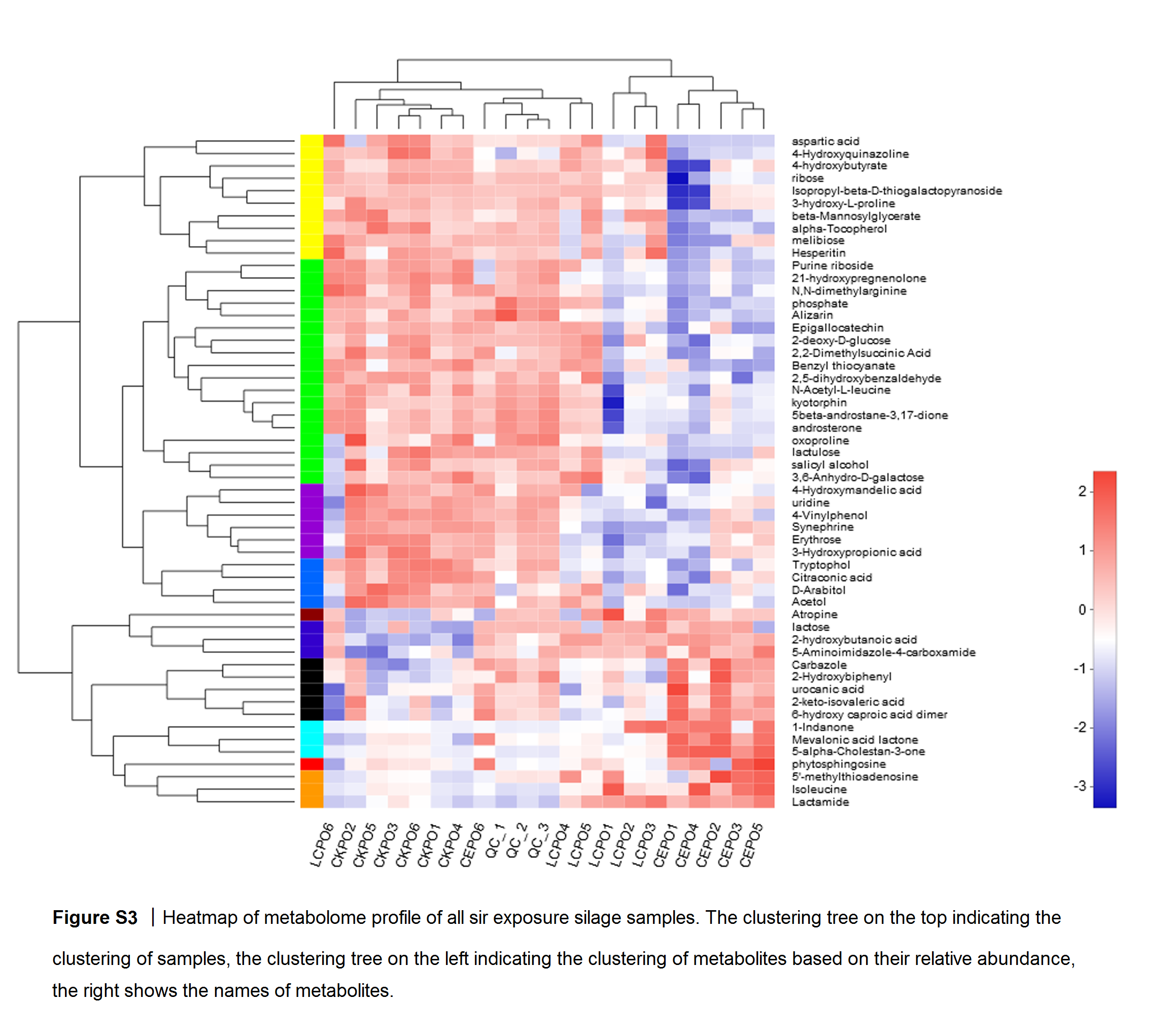

Supplement: Supplementary file 8 [file Image_3.tif]

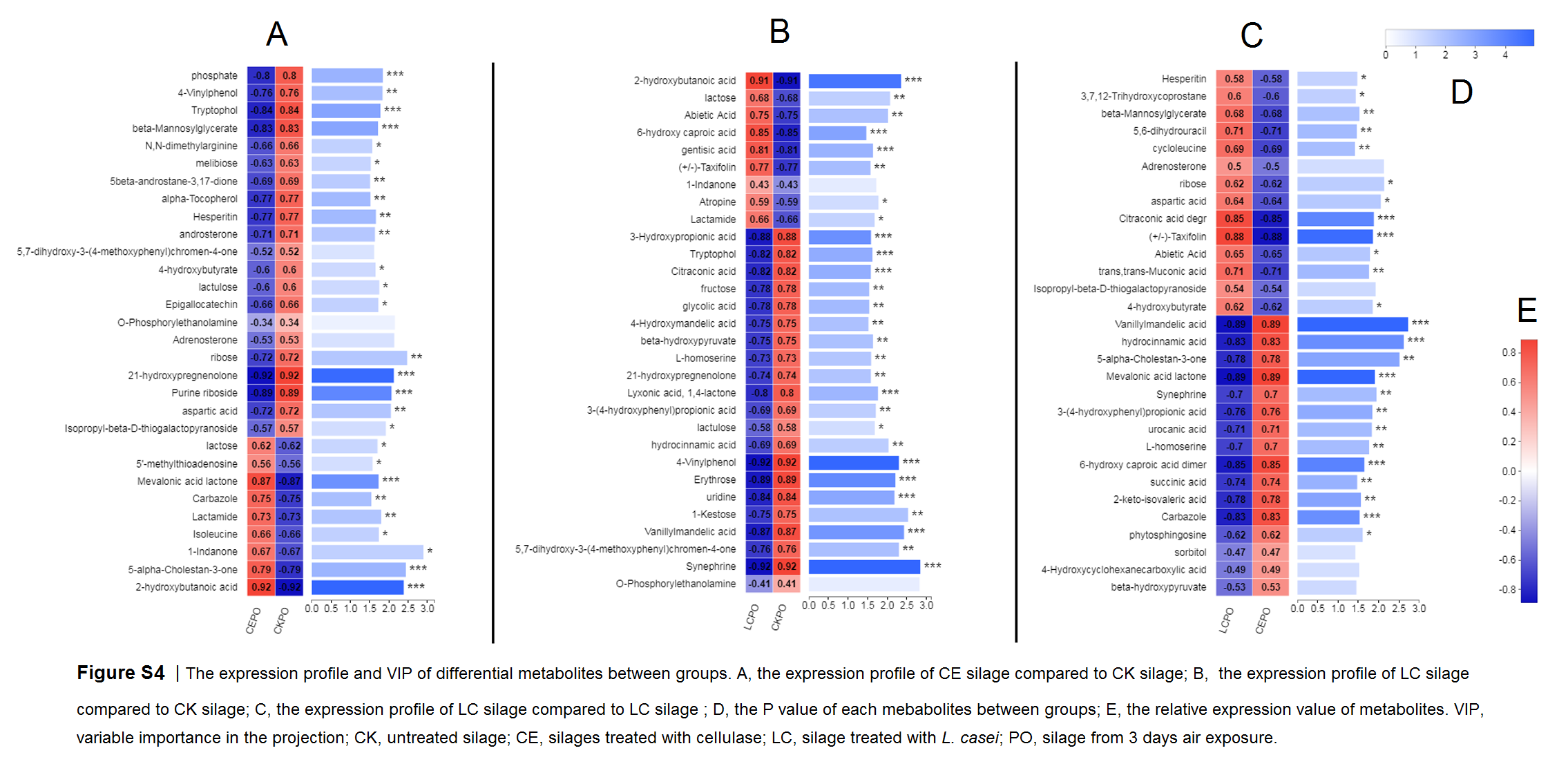

Supplement: Supplementary file 9 [file Image_4.tif]
